# MYB1 transcription factor is a candidate responsible for red root skin in radish (*Raphanus sativus* L.)

**DOI:** 10.1371/journal.pone.0204241

**Published:** 2018-09-21

**Authors:** Gibum Yi, June-Sik Kim, Jeong Eun Park, Hosub Shin, Seung Hwa Yu, Suhyung Park, Jin Hoe Huh

**Affiliations:** 1 Department of Plant Science, Plant Genomics and Breeding Institute, and Research Institute of Agriculture and Life Sciences, Seoul National University, Seoul, Korea; 2 Interdisciplinary Program in Agricultural Genomics, Seoul National University, Seoul, Korea; 3 Department of Horticultural Crop Research, National Institute of Horticultural and Herbal Science, Rural Development Administration, Jeonju, Korea; Chungnam National University, REPUBLIC OF KOREA

## Abstract

Root skin color is one of the economically important traits in radish (*Raphanus sativus*), and the pigmentation in red skin varieties is largely attributable to anthocyanin accumulation. Pelargonidin was found as a major anthocyanin pigment accumulated in the sub-epidermal layer of red radish roots. In the 20 F_2_ population generated from the F_1_ with red root skins, root skins with red and white colors segregated in a 3:1 ratio. Additionally, a test cross between a red F_3_ individual and a white skin individual gave rise to 1:1 segregation of red and white, indicating that the root skin color of radish is determined by a single locus and red color is dominant over white. We performed association mapping for root skin color using SNPs obtained from RNA-seq analysis. Segregation analysis on the 152 F_3_ test-cross population revealed an RsMyb1 transcription factor as a candidate gene to determine root skin color. A PCR marker based on the polymorphism within 2 kb of RsMyb1 was developed and tested on 12 and 152 individuals from F_2_ and F_3_ test cross populations, respectively, and red and white root skin colors were completely distinguished corresponding to the genotypes. Expression levels of *RsMyb1* in red or purple root cultivars were significantly higher than in white root cultivars. These findings suggest that RsMyb1 is a crucial determinant for anthocyanin biosynthesis in radish roots, and the molecular marker developed in this study will be useful for marker-assisted selection for red skin individuals at early seedling stages.

## Introduction

Radish (*Raphanus sativus* L., 2*n* = 18) belongs to the Brassicaceae family, within the tribe Brassiceae. Radish is a root vegetable that is grown and consumed worldwide. Its seedlings, leaves, and siliques are also used as fresh vegetables. Extensive breeding efforts have produced radish cultivars with diverse morphology, varying in root size, shape, color, and growing season, etc. Among them, the exterior root color, which varies from white to black, pink to red and purple, and yellow, is an interesting phenotypic character of *R*. *sativus*. Due to its agronomic importance and accessibility, the exterior root color has been observed and studied for a long time. The major red pigment accumulated in radish root skins is a pelargonidin- or cyanidin-type anthocyanin that is found within the cyanoplasts in the epidermis and in several outer layers of the cortex [1, 2]. Besides major substances as color pigments, anthocyanins are well known for their health benefits, such as antioxidant activities and anti-obesity effects, which could add value to radish [3].

Previous genetic analyses showed that a single or multiple loci may control root skin color. As reviewed by Yarnell [4], several reports have demonstrated that red is dominant over white, with a genetic difference associated with the *R* locus [2, 5]. Mostly, a cross between red and white produce red, purple, and white root skin progenies, with the ratio of 1:2:1. A cross between the radish accessions with specific *R* locus alleles gave rise to progenies with a segregation ratio of 3:1 for red and white [2], albeit some allelic combinations produced progenies with complicated segregation ratios with variable root skin colors [2, 5]. For other root skin colors such as yellow and black, multiple loci have been proposed to be involved [4], but none of these genetic models to determine root skin color is yet supported by strong molecular evidence.

Transcriptional regulation of anthocyanin biosynthesis and its accumulation have been relatively well characterized in many plant species including maize, petunia, and *Arabidopsis*. Several transcription factors are known to regulate anthocyanin biosynthesis in many plant species, in which three types of transcription factors Myb, bHLH, and the WD-repeat proteins play important regulatory roles. For example, *C*, *R*, and *Pl* genes have been cloned and characterized for anthocyanin biosynthesis in maize [6]. PAP1, PAP2, myb113 and myb114 are identified as functional homologs of maize counterparts in *Arabidopsis*, which are also involved in anthocyanin pigmentation [7]. The homologous genes in other species have been identified based on the sequence similarity, and have been shown to perform a similar function. These transcription factors are also interchangeable among species demonstrated by transgenic studies.

In *Raphanus*, however, the genes controlling root color have not been determined due in part to limited genetic resources. Several studies have been conducted for genetics of root skin color in radish but neither the genes nor molecular mechanisms have been demonstrated. Recently, three radish genomes were published from different research groups [8–10], facilitating molecular studies of gene functions associated with specific traits as well as genome-based systemic breeding of radish in the near future. In this study, we report the simple color inheritance in radish, and the molecular genetic analysis allowed us to identify the putative *R* locus gene. In addition, a molecular marker developed from this study will contribute to marker-assisted molecular breeding associated with anthocyanin pigmentation in radish.

## Materials and methods

### Plant materials

A Chinese commercial F_1_ cultivar *R*. *sativus* cv. Lian Yan No. 1 (Danong Seed Co., Liaonin Sheng, China) with the red skin color was used as a primary material for genetic analysis. The F_1_ was self-pollinated to produce 20 F_2_ population and further self-pollinated to generate F_3_ population. The single F_2_ descendant 35 F_3_ population, which segregated for root skin color, was subjected to RNA-seq. Each F_3_ individual was self-pollinated and the resulting 104 F_4_ plants were planted to confirm the original F_3_ genotype. F_3_ plants were also test-crossed with a recessive homozygote to produce the mapping population (152 individuals, *Rr* × *rr*). Plants were grown in a 10-cm diameter and 6-cm height pot in a growth room at 24°C under 16-hour day condition. For phenotyping, over 10 plants were grown in the field (Suwon, Korea) with conventional culture methods [11]. Currently available domestic commercial radish cultivars were analyzed for their root colors and RsMyb1 expression levels at 4 weeks after planting: KN Red King, KN Ruby King, KN Ruby Ball, and KN Bravo from Kwonnong Seed Co. (Cheongju, Korea), Bordeaux and Jeongwon (Syngenta Korea) for red or purple skin radish cultivars, and Geumbong (Kwonnong), Chungwoon (Syngenta Korea), and Jinjudaepyung (Jinju, a landrace) as white root skin cultivars.

### Microscopy

Hand-sectioned samples were visualized under a stereomicroscope (SteREO Discovery. V12, Carl Zeiss, Germany) equipped with the digital camera (AxioCam MRc, Carl Zeiss, Germany). Sections were stained with toluidine blue O, if necessary, to visualize the cell shapes.

### HPLC analysis

Anthocyanidin profiles of red and white root skins were investigated by HPLC analysis. Root skin was peeled from three homozygous red and white root individuals from F_4_ and pooled, respectively. After grinding the root skin tissues with liquid nitrogen, the red pigment was extracted with a solvent mixture containing acetone (40%, v/v), methanol (40%, v/v), and formic acid (0.1%, v/v). A VDS C-18 column (4.6 × 250 mm, 5 μm, VDS Optilab, Germany) was used with a 10 μL injection volume and 0.8 mL min^-1^ flow rate. Identification of individual anthocyanidins was performed with HPLC and LC-MS as previously described [12].

### RNA-seq analysis

RNA-seq was performed with 31 separately barcoded libraries (8 white and 23 red root skin individuals), which were generated from the total RNA of young leaf tissues. The library construction and sequencing were performed as previously described [13]. On average, 1.5 Gb of raw sequence reads from each library was obtained. Low-quality sequences (< Q20) as well as barcodes and adaptor sequences were trimmed out using Trim Galore v.0.3.7 with the default parameters but ‘—gzip–paired’. The purified reads were subjected to the SNP analysis. The raw sequence data have been deposited in the NCBI/EBI/DDBJ Short RNA Archive (PRJNA347524).

### SNP calling and association mapping

The published radish genome (RadishV1.2.2, radish-genome.org) was adopted as a matrix for mapping of sequence reads [8]. Each purified read sequences were aligned against the radish genome by running STAR v2.4.2a 2-pass alignment [14]. Duplicated reads from individual alignments were marked using Picard Tool (v2.4.1) MarkDuplicates and omitted in the following analyses. The Genome Analysis Toolkit (GATK; v3.5–0) RealignerTargetCreator and IndelRealigner protocols were used for global realignment of reads around indels [15]. SNP calling utilized GATK HaplotypeCaller and GenotypeGVCFs with default settings. The filtering settings to validate the SNPs were as follows: FS > 30, DP > 2000, and QD < 2.0. SNP calling was repeated twice with the GATK BaseRecalibrator protocol to reduce ambiguous SNPs. Finally, a total of 208,469 biallelic SNPs were retrieved from the raw, unfiltered 532,923 SNPs. Before association analysis, ungenotyped markers were imputed using Beagle v4.1 [16]. The association analysis to identify the region linked to the root skin color phenotype was performed with FaST-LMM v2.02, running on the Hardy-Weinberg estimate [17].

### Marker development and segregation analysis

PCR primers to differently amplify specific fragments present in the candidate region of chromosome R7 were designed according to the intergenic region sequences and used to detect polymorphism. Polymorphic PCR bands were used to detect recombination in the F_2_ and F_3_ test-crossed populations (*Rr* × *rr*). The primers of the PCR markers are listed in Table 1. PCR amplicons were visualized with EtBr on a 2% agarose gel.

**Table 1:** PCR markers for radish root skin color.

Locus	Primer sequence (5'–3')	Tm^a^ (°C)	Amplicon size (bp)^b^
**R7-7.97**	ACTCGACGTCTGCTTGGAAT	55	578
	TTCCTCCTGCATAACGTTCC		
**R7-8.47**	CTCAACATAGCGCATGGAAA	55	582
	CGTGTGATGGACCAACTTTTT		
**R7-9.36**	GGCCTTTAGATAGAAATCTATAGCTACCGA	57	394
	GTACCGATCATCACCGTCCTC		
**R7-12.39**	ATCGAACATCTATGGCTCGTTT	55	745
	GGTCTTCCCAGACTTTTGTGTC		
**R7-15.29**	GGCTGCATCGTCGATAGAAT	55	572
	CCAACAAGGGTTGTGCTTCT		

^a^ The temperature used for PCR reaction in this study.

^b^ Amplicon size is based on WK10039 genome sequence.

### Quantitative real-time PCR (qRT-PCR) analysis

Four red and four white individuals from F_3_ test-crossed population (*Rr* × *rr*), were used for qRT-PCR. Root skins were peeled and frozen with liquid nitrogen. RNA was extracted with RNeasy Plant mini kit (Qiagen, Germany) and treated with On Column DNase (Qiagen, Germany), according to the manufacturer’s protocol. cDNA was synthesized from 1 μg RNA, with Superscript III reverse transcriptase (Invitrogen, USA). qRT-PCR was performed using the Rotor-Gene Q 2plex HRM platform and QuantiFast SYBR Green PCR kit (Qiagen, Germany). For testing red and white root skin cultivars, whole roots including the hypocotyl, which were grown in a growth room, were used for RNA extraction and further qRT-PCR. Primers used for qRT-PCR are listed in Table 2.

**Table 2:** PCR primers used for qRT-PCR.

Locus	Primer sequence (5'–3')	Predicted function	Tm (°C)	Expected size (bp)
**R7_9141477_9142640(+)**	CTCTGATGGCTAACCCTTACG	binding partner of ACD11 1-like (AT1G67950)	60	94
	CCTCCATTCTCAGCTCTAACG			
**R7_9145761_9147238(-)**	TCAGGTATGATCAACGGCAC	heat stress transcription factor A-8 (AT1G67970)	60	126
	CCAGAAGGCACCATCAGAG			
**Rs388430**	CGTCCAAAGGGTTGAGAAAAG	transcription factor MYB114-like	60	141
	GTCTACAACTCTTCCTGCACC			
**Rs386970**	GAATGAAATGGTCGCCTTGTG	isoflavone reductase homolog P3	60	136
	CGAGTGGTTTATGGCGAGAAG			
**Rs386960**	ACCAAACATTCGCTCTCGTC	isoflavone reductase homolog P3-like	60	118
	TCCTTGTCGCTTAAATCTCCG			
**Rs386430**	CAACAACATGGCCCTGGAAA	myb-related protein 306-like	60	125
	GTGAAATTTCCTCGTTTGATCCCT			
**Rs386290**	GTTTACATCAGCGAACACGG	transcription repressor MYB6-like	60	97
	TCAGCCACCTTAATCTACAACTC			
**R7_11797046_11798366(-)**	CTCCATCTAACAGGCTCCAAAGT	transcription factor MYB114-like	60	89
	AGCTGCACTCTTTCCAGAAG			

## Results

### Root skin color is determined by a single dominant gene

**Fig 1:**
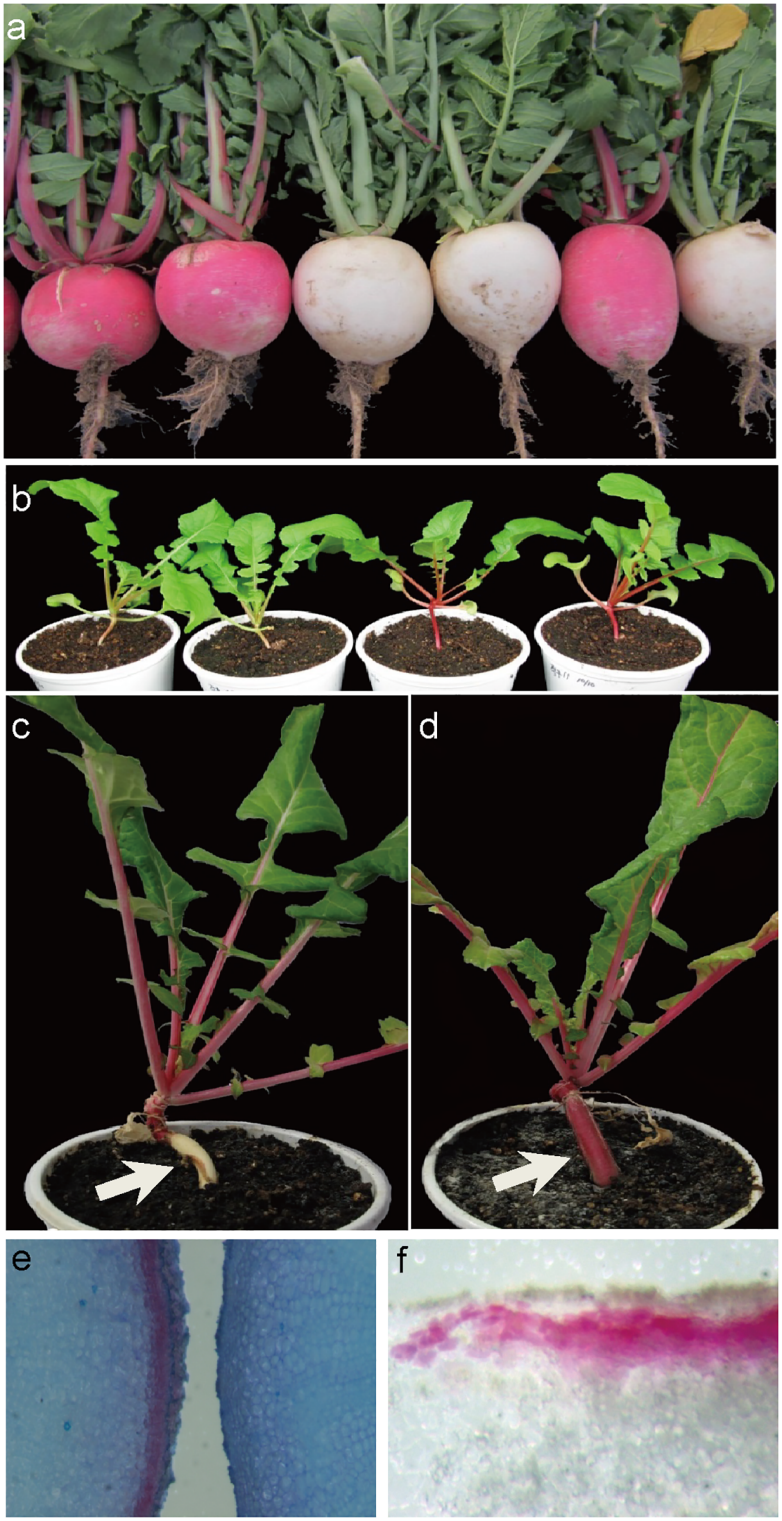
Phenotypes of red and white root skin radishes. (a) Root skin color phenotypes of segregating F_2_ individuals grown in a field. (b) Hypocotyl skin colors of F_3_ plants at 21 days after planting (DAP). Note that in many instances, the hypocotyl and petiole colors are already separated but they are not always identical to the root skin color. (c, d) White and red root skin colors of F_3_ plants at 120 DAP, when root and hypocotyl thickening is in progress (e) Stereomicroscopic images of transverse sections of the red (left) and white (right) roots stained with toluidine blue O. Samples are from the plants shown in (c) and (d). (f) Accumulation of anthocyanin pigments in the 2–4 layers of the subepidermal cells of red colored root (from c).

The radish plants were grown both in a growth room and in a field. The hypocotyl and root began radial expansion from 4 weeks after planting, and almost at the same time, root skin color became visible (Fig 1a). The population in this study had two different types of hypocotyls, red and white (Fig 1b). Red and white root skins were clearly distinguished under both growing conditions (Fig 1a and 1c). However, root skin color and hypocotyl color were not the same in many individuals, suggesting that pigmentation of root skin and hypocotyl is regulated by different processes.

**Fig 2:**
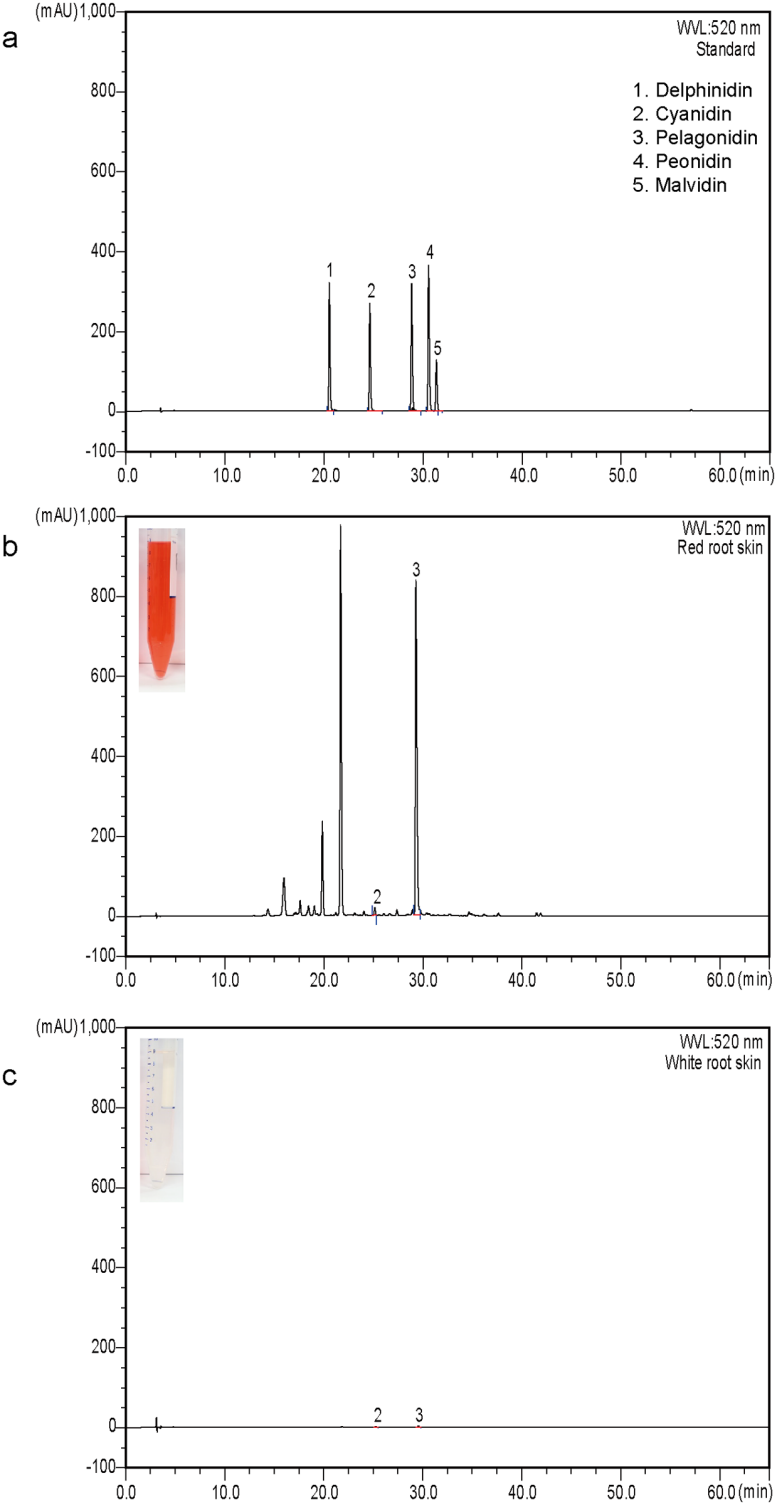
Pelargonidin is a major anthocyanidin in red root skin. HPLC analysis with standards (a), and root skin extracts of red (b) and white roots (c). Pictures for methanol extracts from the red and white root skins were depicted in inlets in (b) and (c), respectively.

We found that red pigments were accumulated in 2–4 layers of subepidermal cells as observed under a stereo microscope (Fig 1e and 1f). The red pigment was identified as anthocyanins as previously reported [18, 19]. Major anthocyanins were analyzed by HPLC with known standards (Fig 2), and all major peaks present in the red root skin extract were further confirmed by LC-MS (S1 Fig). Two anthocyanidins were detected as major pigments, in which pelargonidin was the most abundant comprising 98% of total anthocyanidins (S2 Fig). A small amount (2%) of cyanidin was detected in red root skin extracts.

The F_1_ Chinese commercial cultivar was self-fertilized and the exterior root color was found to segregate 3:1 for red and white in the F_2_ population (Table 3). Self-pollinated progenies of white root skin radish all displayed white colors, whereas the descendants from red showed either all red or a 3:1 segregation ratio for red and white. The 3:1 segregation ratio was consistently observed in the F_2_, F_3_ and F_4_ segregation populations, in a total of 159 plants (P > 0.492, Table 3). Moreover, when F_3_ heterozygous red root skin individuals (*Rr*) were test-crossed with white root skin (*rr*) individuals, the descendant showed a 1:1 (red: white) segregation ratio (n = 152, P = 1). From this analysis, we concluded that a single dominant gene controls the red exterior root pigmentation in these populations.

**Table 3:** Root skin color segregation.

Population	Red	White	Total	χ^2^	P
F_2_	14	6	20	0.267 (3:1)	0.6056
F_3_ (*Rr* × *Rr*)	25	10	35	0.238 (3:1)	0.6256
F_4_ (*Rr* × *Rr*)	84	20	104	1.846 (3:1)	0.1742
F_3_ test-cross (*Rr* × *rr*)	76	76	152	0.000 (1:1)	1.0000

### Colors of other vegetative tissues are associated with root skin color

Accumulation of anthocyanins was observed in most of the tissues including the hypocotyl, petiole, stem, and petal (Fig 1a–1d). Plants with a red root skin tend to display red colors in the hypocotyl and the petiole but not in the petal (Table 4). In order to determine the relationship of coloration in different tissues, the colors of the other parts of radish plants were observed and categorized into red and white. The petiole color was sometimes difficult to determine because medium reddish hues were observed. In that instance, the color of the adaxial side of the petiole was used as a color indicator. In total, 16.6% and 9.9% F_3_ test-cross progenies displayed color differences between root skin and hypocotyl colors, and between root skin and petiole colors, respectively; and 18.4% showed discrepancy between the hypocotyl and petiole colors (Table 4). Two possible explanations could be proposed. First, two corresponding genes are closely linked. Second, the pigmentation of vegetative tissues including root skin, hypocotyl, and petiole, is regulated by the same gene. However, 47.6% of individuals had different root skin and petal colors (Table 4), suggesting different genetic regulations are involved in reproductive tissue coloration. The mismatch between the root exterior and hypocotyl and root skin color became evident within 4 weeks after planting, indicating the need for a molecular marker that can select red skin root at an earlier stage.

**Table 4:** Relationships among tissue colors.

		hypocotyl		petiole		petal	
		R	W	R	W	R	W
root	R	75	0	75	0	40	32
	W	25	51	15	61	37	36
hypocotyl	R			82	19	55	39
	W			9	42	20	29
petiole	R					47	39
	W					28	29

### Association mapping by RNA-seq identifies the R locus

**Fig 3:**
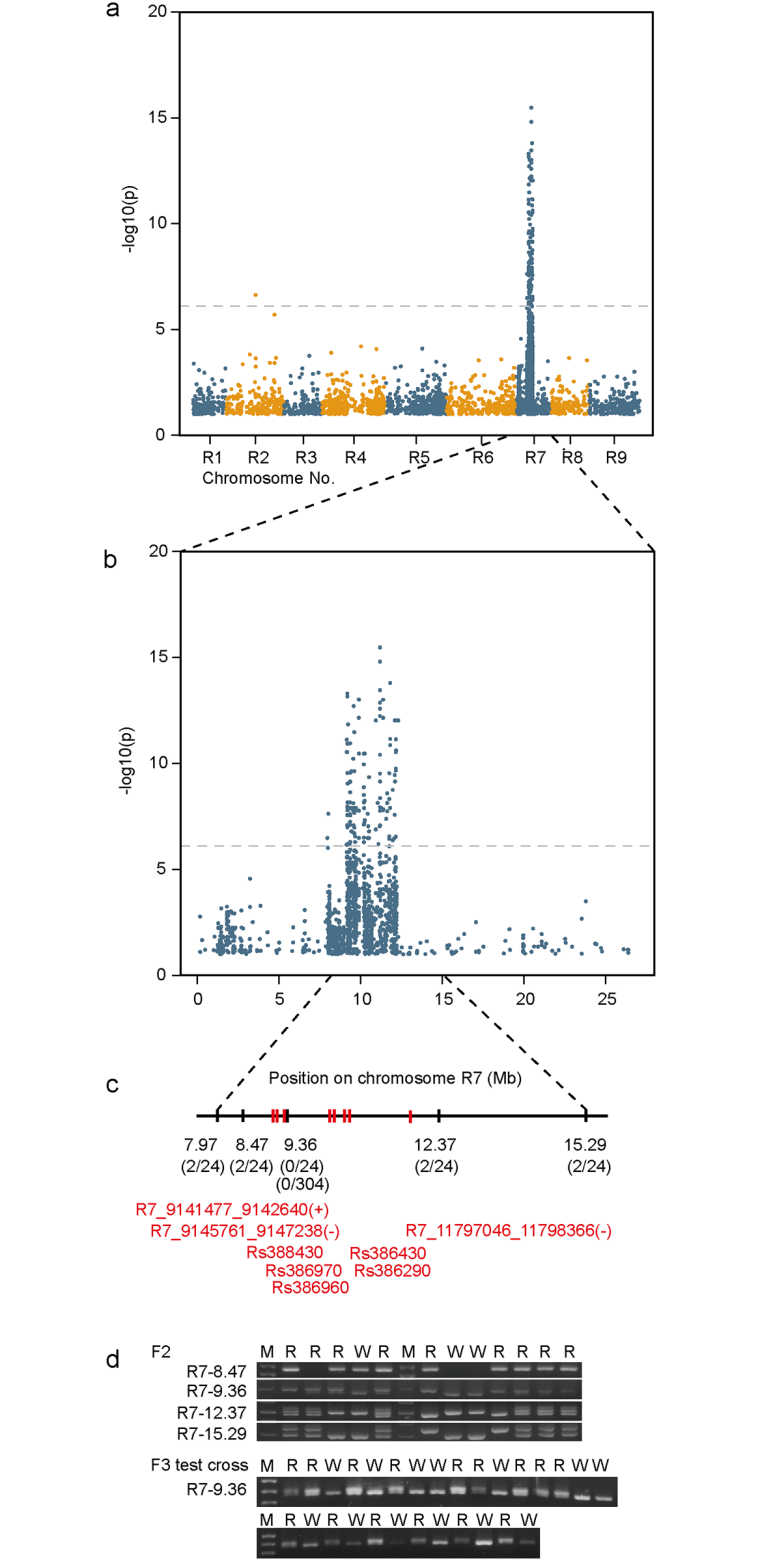
Genome-wide association analysis and genetic linkage analysis of F_3_ individuals indicate the red root skin color locus is on chromosome R7. (a) Manhattan plot showing -1og_10_ *P*-values of 208,469 SNPs associated with root skin color relative to their genomic positions on the x-axis. The dotted red line indicates 5% significance with Bonferroni correction. (c) Relative positions of PCR markers (black bars) and candidate genes (red bars) on chromosome R7. Note that the marker R7-9.36 and a strong candidate were proximally located within 10 kb. (d) Twelve F_2_ individuals were genotyped with PCR makers, which were designated and named after their chromosomal positions. The R7-9.36 Mb marker was further applied to 152 individuals of F_3_ test-cross population. The numbers of recombinants/chromosome tested are presented in parentheses (c). M, size marker; R, red; W, white root skin individuals.

A single F_2_ descended F_3_ population, which segregated for root skin color, was subjected to association studies. RNA was extracted from young leaf tissues from 23 red (*RR* or *Rr*) and 8 white (*rr*) F_3_ radish individuals and used for RNA-seq. We used leaf tissues for RNA-seq primarily to identify SNPs between different root color individuals without damage to the root system. By detecting SNPs from the RNA-seq data, we successfully identified a group of SNPs that cosegregated with the root skin color, in which the first group was likely to have SNPs specific to dominant (*RR*) or heterozygous (*Rr*) alleles for red, whereas the second group was predicted to have SNPs specific to the recessive (*rr*) alleles for white. A total of 208,469 SNPs were scattered throughout the entire chromosomes of radish genome (Fig 3a). Notably, a single major peak associated with root skin color was identified on radish chromosome R7 (Fig 3b), and thus, it is highly plausible that this region (9.11–11.78 Mb) contains the *R* locus responsible for root color determination. A total of 339 genes involved in various functional categories are annotated in this region. As described below, we further verified their association with the root color phenotype by performing cosegregation analysis with corresponding molecular markers, and assessed their possible contribution to root skin color in relation to the putative functions of these genes.

### RsMyb1 is located in the region of chromosome R7

In an attempt to identify the putative *R* locus gene, we first confirmed the association mapping results by developing molecular markers specific to 9.11–11.78 Mb region of chromosome R7. A total of 32 PCR markers were developed based upon bialleleic SNPs and indels, and seven of them were found polymorphic (Fig 3c and Table 1). Five markers were co-dominant amplifying allele-specific fragments, which greatly improved the quality of recombination analysis. Recombination frequencies between the putative *R* locus and the loci detected by PCR markers were analyzed for 12 F_2_ individuals. The putative region was narrowed down to 8.47–12.37 Mb estimated by observed recombination frequency (Fig 3c). Remarkably, R7-9.36 completely cosegregated with the root skin color phenotype in F_2_. Further analysis revealed that R7-9.36 had a perfect cosegregation with the root skin color in 152 individuals of F_3_ test cross population (*Rr* x *rr*) (Fig 3c). This indicates that the *R* locus is tightly linked to R-7.36, and therefore, nearby coding genes are the most likely candidate for the *R* locus gene determining the radish root color. Most notable among several coding genes in this region is a MYB1 transcription factor (called RsMyb1 hereafter), whose contribution to anthocyanin biosynthesis has been reported in a few plant species [6, 7].

### RsMyb1 is upregulated in red root skin

**Fig 4:**
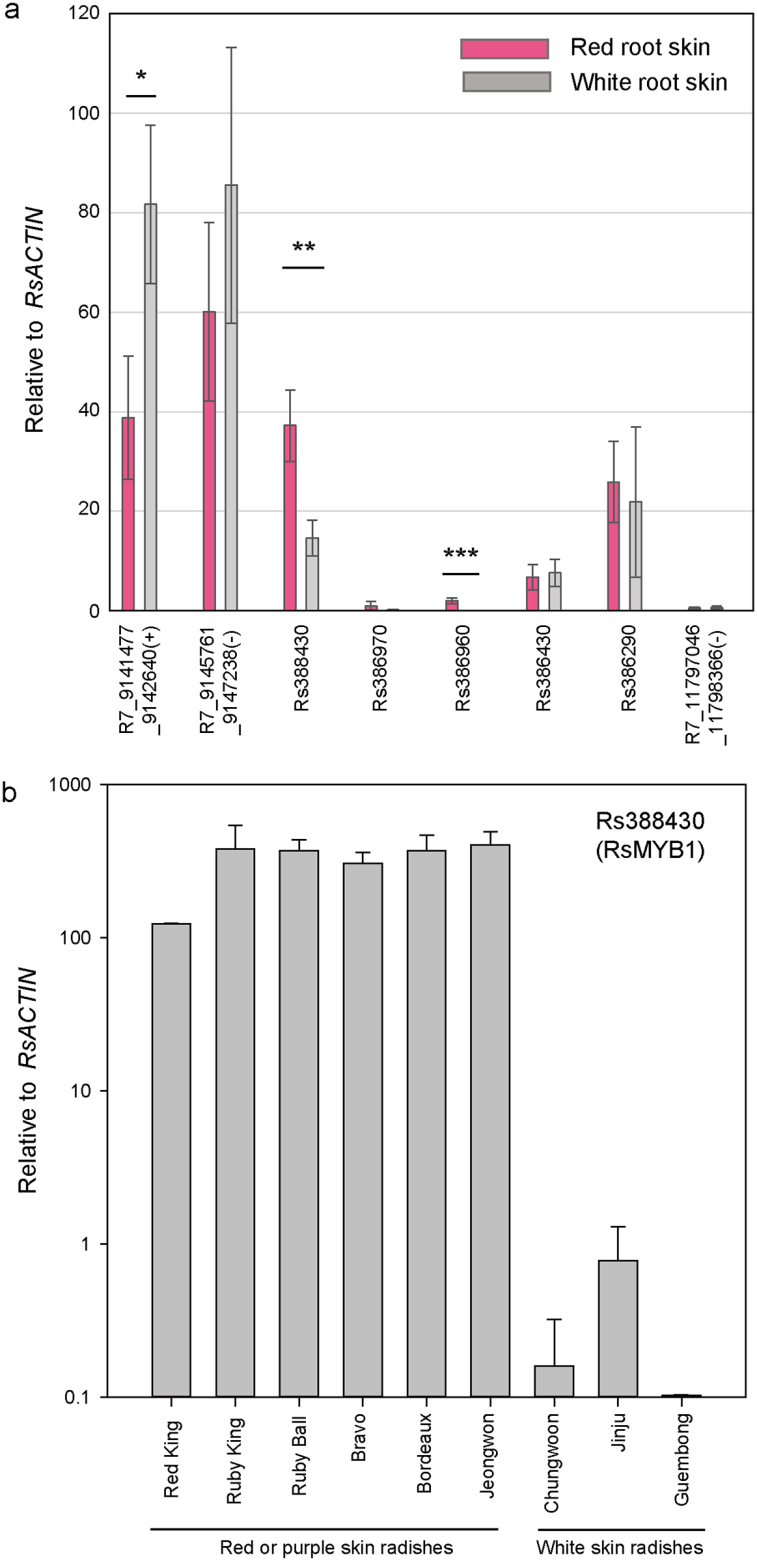
Transcription levels of *RsMyb1* and other candidate genes in red and white root skins. (a) Transcription levels were measured by qRT-PCR and presented relative to *RsACTIN*. Positions on chromosome R7 and predicted functions of the genes are as follows: *R7_9141477_9142640*(*+*) (binding partner of ACD11 1-like), *R7_9145761_9147238*(*-*) (heat stress transcription factor A-8), *Rs388430* (R7_9348525_9349979(+), transcription factor MYB114-like) *Rs386970* (R7_10205078_10206725(-), isoflavone reductase homolog P3), *Rs386960* (R7_10214323_10216096(-), isoflavone reductase homolog P3-like), *Rs386430* (R7_10493232_10495210(-), myb-related protein 306-like), *Rs386290* (R7_10584636_10586808(-), transcription repressor MYB6-like), *R7_11797046_11798366(-)* (transcription factor MYB114-like). * p < 0.05, ** p < 0.01, ***p < 0.005. Bars indicate means ± standard deviation from three biological replicates. (b) Expression level of *Rs388430* (*RsMyb1*) was analyzed with qRT-PCR in six red/purple and three white root radish cultivars as indicated. *Rs388430* expression was consistently higher in red or purple root cultivars than in white root cultivars. Error bars indicate means ± standard deviation from the three biological replicates.

Although we successfully identified SNPs associated with root skin colors from the RNA-seq analysis, the current transcriptome data are not relevant to the expression profiles explaining the root skin color phenotype because sequencing was conducted with leaf tissues. In order to assess the contribution of the coding genes present in region 8.47–12.37 Mb of chromosome R7 to root skin color, we have carefully chosen candidate genes according to the following criteria: first, differential expression levels between red and white root radishes (S3 Fig); second, root-specific expression reported in the Radish Genome Database (radish-genome.org); and third, regulatory functions for anthocyanin biosynthesis reported in other plant species. Eight genes were selected and subjected to further analysis to verify their contribution to root skin color as a putative *R* locus gene (Table 2). Their expression levels were determined by qRT-PCR (Fig 4a), and three of them were shown to have significant differential expressions between red and white root tissues. A putative RNA-binding protein (R7_9141477–9142640) was highly expressed in white roots compared to red roots, whereas both MYB114-like transcription factor (Rs388430, called RsMyb1 hereafter) and isoflavone reductase (Rs386960, called RsIFR1 hereafter) were expressed at higher levels in red root tissues than in white ones (Fig 4a). Phylogeny analysis revealed that RsMyb1 was clustered together with the R2R3 motif transcription factors such as MYB113, MYB114, PAP1 and PAP2, all of which are known to be involved in the regulation of anthocyanin biosynthesis in other plant species (S4 Fig) [7]. Further expression analysis on red and white root radish cultivars also revealed that *RsMyb1* was expressed at significantly higher levels in red root cultivars than in white root cultivars (Fig 4b). This strongly suggests that *RsMyb1* expression is specific to red radish roots and determines the external root color of radish by regulating anthocyanin biosynthesis. RsMyb1 was cloned from red and white root F_3_ individuals and their sequences were compared each other. *RsMyb1* genes isolated from red and white root radishes (called *RsMyb1-R* and *RsMyb1-W*, respectively) are predicted to encode the polypeptides with the same size (249 amino acids) but with differences in amino acid composition at 18 positions (S5 Fig). However, these amino acid differences are unlikely to cause differential activities between RsMyb1-R and RsMyb1-W considering the conserved structure of RsMyb1 compared to other MYB transcription factors. Moreover, RsMyb1-R is more similar to RsMyb1 isolated from white root WK10039 cultivar, and RsMyb1-W is similar to RsMyb1 of red root Bordeaux (S5 Fig). Comparison of the nucleotide sequences outside the *RsMyb1* coding region also revealed 13 indels and 14 SNPs between *RsMyb1-R* and *RsMyb1-W* alleles (data not shown), suggesting that these differences in *cis*-element might be responsible for differential expression of *RsMyb1* in red and white root radishes, although we cannot completely rule out the possibility that polymorphism in *RsMyb1* gene itself is responsible for differential pigmentation in red and white radish roots.

Tissue-specific expression analysis revealed that *RsMyb1* is expressed in all seedling stage tissues such as leaves, hypocotyls and radicle roots but expressed at significantly lower levels than in developing roots (S6 Fig). These observations are consistent with the expression database showing that *RsMyb1* is predominantly expressed in roots during 3–9 weeks after planting with maximum expression at 3 weeks (radish-genome.org). Despite the relatively low expression levels in all seedling tissues, *RsMyb1* is generally expressed at higher levels in most tissues of red root radish than in white radish (S6 Fig). Moreover, the expression level of *RsMyb1* in the root skins of red root cultivars was found to be 158–518 times higher than in white roots of Jinju, which displayed the highest *RsMyb1* expression among white root radish cultivars (Fig 4b). *RsIFR1* was also found to be expressed mostly in roots but its expression level did not correlate with the root skin color (S7 Fig), suggesting that RsIFR1 is unlikely to determine the pigmentation of radish roots. All these findings strongly suggest that RsMyb1 is a strong candidate for the *R* locus gene determining the root skin color of radish.

## Discussion

Root skin color is an interesting phenotype in radish, varying from white, yellow, red, and purple to black among others. Conventional genetic studies have reported possible modes of inheritance of radish root skin color [4]. In 1923, Frost reported that a cross between red and white root radishes produced the F_1_ plants with purple or violet-pink roots, suggesting not only that purple color was dominant over red and white colors but also that white radish cultivars carried a purple gene since all the F_1_ plants were purple or violet [5]. This study represented the recessive “red” gene by *r*, and its dominant “wild-type” (purple) allelomorph by *R* [5]. Consistently, it was also reported that a cross between the Long Red (red) and Icicle (white) cultivars produced the F_1_ plants with entirely violet to violet-purplish roots, and subsequently the F_2_ plants with a segregation ratio of 1:2:1 for red, violet, and white roots [2]. Similar results were obtained from another cross between Early Red (red) and Early White (white) [2]. These observations suggested that the root exterior color might be controlled by a single locus involving multiple alleles, the combination of which determined the pigmentation in the root exterior [2, 5]. However, a later study found the genetics of radish root coloration more complicated with multiple factors involved [20]. This study proposed that radish root color is determined by the three factors *R*, *B*, and *Y*, by which the probable genotypes and root color phenotypes are assumed as follows: *RRBByy*, purple; *RRbbyy*, red; *rrBByy*, white; *rrBBYY* or *rrBbYY*, yellow; and *rrBBY*^*b*^*Y*^*b*^, black. In the cross between red and white root cultivars, the F_1_ was always purple, and the F_2_ showed a segregation of 9 purple: 3 red: 4 white. This indicates that the dominant *R* is responsible for red pigmentation, whereas *B* results in white by itself, but in conjunction with *R*, produces purple pigments [20].

In our study, the Chinese commercial F_1_ cultivar Lian Yan No. 1 was used as a starting material, and a cross between homozygous red and white root individuals always produced red-colored roots. Its progeny displayed a segregation ratio of 3:1 for red and white, and a testcross of red-colored heterozygous individual with white one produced 1 red: 1 white. These observations strongly suggest that root pigmentation, in this case, is primarily governed by a single genetic factor that distinguishes between red and white colors, in which red color is completely dominant over white. When fitted to the Tatebe model, it is very likely that Lian Yan No. 1 used in this study is heterozygous for *R* (*Rr*) but homozygous recessive for both *B* and *Y* (*bbyy*), since no colors other than red and white were observed in any cross. According to the previous literature, the radish rook skin color appears to be largely determined by the genetic factors [1, 2, 5]. Contribution of environmental factors such as sunlight and abiotic stress are also important for anthocyanin biosynthesis, but the skin pigmentation in radish root is mostly qualitative even though some variations exist for the degree of coloration in this study.

We identified a total of 208,469 SNPs from RNA-seq and performed association mapping for root skin color in 23 red and 8 white root individuals. A genomic region located on radish chromosome R7 was revealed to be highly associated with the root skin color phenotype, and we successfully identified a panel of candidate genes in the corresponding scaffold. Most notable among them is *RsMyb1*, encoding an R2R3-MYB transcription factor which is also known as an important regulator of anthocyanin biosynthesis in many plant species [21–23]. Recently, *RsMyb1* was isolated from a cDNA library of purple root radish cultivar ‘Bordeaux’, and shown to ectopically upregulate anthocyanin biosynthesis when expressed in Arabidopsis and tobacco [24]. This suggests that RsMyb1, when activated in the root skin, is a key factor that regulates the anthocyanin biosynthesis pathway, eventually determining the root skin color.

Utilizing the SNPs and indels identified in the scaffold spanning 9.11–11.78 Mb on chromosome R7, we developed several PCR markers (Table 1), and the following linkage analysis showed that the R7-9.36 marker was completely cosegregated with the root skin color phenotype in 12 F_2_ and 159 F_3_ testcross individuals (Fig 3). Considering the positions of this marker and *RsMyb1* and their tight linkage to red/white root color phenotype, it is highly convincing that *RsMyb1* is the *R* locus gene that determines the root skin color in radish.

We found that *RsMyb1* was highly upregulated (>100 fold than in white root cultivars) in all red or purple root radish cultivars examined (Fig 4), suggesting that RsMyb1 a common key factor for root skin color in other radish cultivars as well. However, while upregulation of *RsMyb1* critical for anthocyanin biosynthesis in radish roots, pigmentation in other parts appears to be regulated by different mechanisms. For instance, F_2_ and F_3_ progenies of Lian Yan No. 1 showed no strong correlation between root coloration and that of other tissues. In addition, even white root cultivars sometimes developed red colors in hypocotyl, leaves, and flowers, displaying little correlation with root colors. Moreover, both red and white root radish cultivars have similar RsMyb1 coding sequences but their expression levels significantly differ, indicating that a difference in tissue-specific anthocyanin pigmentation is largely determined at the transcription level due probably to different *cis* element structures, which requires further investigation.

HPLC analysis revealed that red radish root skins synthesized a large amount of pelargonidin (~98% of total anthocyanidin), with very little cyanidin (<2% of total anthocyanidin). Such ratio of pelargonidin and cyanidin was also reported for Chinese red root cultivar Hong Feng No. 1, which has a skin color and root shape similar to those of Lian Yan No. 1 [25]. Every tissue we observed displayed red or pink color rather than purple. Notably, purple root radish cultivars Benikanmi and Bordeaux were reported to accumulate prominently more cyanidin in the root than red ones such as Man Tang Hong and Hong Feng No. 1 which have red root skins with high levels of pelargonidin [19, 25, 26]. This suggests that the ratio of pelargonidin and cyanidin affects the visualization of root color, with pelargonidin and cyanidin being responsible for red and purple colors, respectively. It was proposed that the *H* locus is involved in radish root color determination distinguishing between red and purple (or violet) [1]. Pelargonidin and cyanidin differ only for the presence (cyanidin) or absence (pelargonidin) of a hydroxyl group at 3’ of ring B of flavonoid. It is notable that flavonoid 3’ hydroxylase (F3’H) catalyzes 3’ hydroxylation of dihydrokaempferol, a precursor of pelargonidin, into dihydroquercetin leading to the biosynthesis of cyanidin (S2 Fig). Therefore, the gene encoding F3’H will be likely a putative *H* locus gene that distinguishes between red and purple pigmentation in radish roots.

Finally, this study demonstrates the feasibility of association mapping using RNA-seq data to identify the candidate gene responsible for a trait of interest. Recently available radish genome information will further facilitate the isolation of the genes involved in many interesting traits, and the molecular markers developed in this study will be highly useful for genome-assisted breeding of radish cultivars with high anthocyanin pigmentation.

## Supporting information

S1 Fig LC-MS qualification of anthocyanin peaks in red root skin samples. LC chromatogram of anthocyanin standards (a) and red root skin radish samples (b). (c) Mass spectrum of cyanidin at 8.132 min. (d) Mass spectrum of pelargonidin observed at 9.022 min. Note that other high-intensity peaks were determined not to be anthocyanins.
(TIF)

S2 Fig Anthocyanin biosynthesis pathway. Enzymes for each step were abbreviated as indicated in the box. Two major anthocyanins pelargonidin 3-glucoside and cyanidin 3-glucoside present in radish roots are presented in colored boxes.
(TIF)

S3 Fig Relative expression levels of annotated genes in 23 red vs 8 white root skin individuals. The FPKM and fold change averages were plotted. Significantly up- and downregulated genes were selected by the cutoff determined by Welch’s *t-*test and FDR < 0.05, and colored as indicated.
(TIF)

S4 Fig Phylogenetic tree of MYB family genes in radish and *Arabidopsis*. Red lines indicate the branch for anthocyanin accumulation regulators. RsMYB1 is denoted in red. HOS10, high response to osmotic stress 10; PAP1, 2, production of anthocyanin pigment 1, 2; TT2, transparent testa 2; AS1, asymmetric leaves 1; CDC5, cell division cycle 5; DUO1, duo pollen 1; AT4G17780, F-box and associated interaction domains-containing protein.
(TIF)

S5 Fig Alignment of RsMYB1 sequences of white and red root radishes. Translated RsMYB1 amino acid sequences were aligned to RsMYB1 from Bordeaux (AKM95888), WK10039 (Rs388430) and AtMYB113 (AT1G66370) by CLUSTAL-OMEGA. Green lines indicate the R2R3 domains of MYB DNA-binding proteins. Shaded boxes indicate putative DNA binding sites [27].
(TIF)

S6 Fig *RsMyb1* transcription levels in five different tissues at seedling (14 DAP) and adult (200 DAP) stages. qRT-PCR was performed with three biological replicates for red and white root skin radishes, respectively. Data are means ± SD. * p < 0.05.
(TIF)

S7 Fig Expression of Rs386960 in six red and three white skin radish cultivars. (a) qRT-PCR analysis of Rs386960. Error bars indicate the standard deviation of the mean from three biological replicates. (b-f) Four-week-old roots of some radish cultivars used for qRT-PCR in Fig 4b and (a).
(TIF)
