# Supplementary figures and images for: MYB1 transcription factor is a candidate responsible for red root skin in radish (Raphanus sativus L.)
Source: PLoS One. 2018 Sep 21;13(9):e0204241. doi: 10.1371/journal.pone.0204241 (PMC6150496; doi:10.1371/journal.pone.0204241)

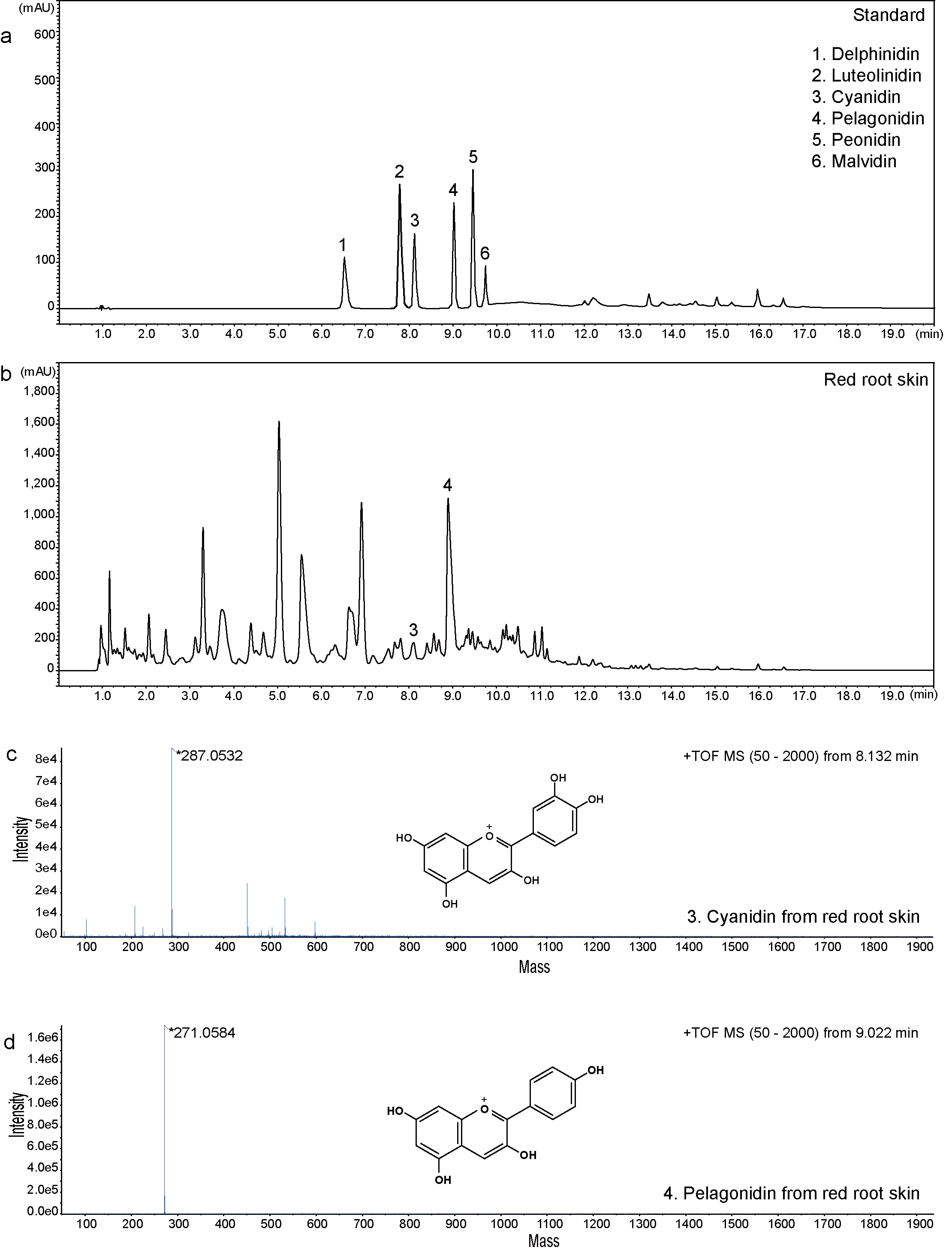

Supplement: S1 Fig — LC chromatogram of anthocyanin standards (a) and red root skin radish samples (b). (c) Mass spectrum of cyanidin at 8.132 min. (d) Mass spectrum of pelargonidin observed at 9.022 min. Note that other high-intensity peaks were determined not to be anthocyanins. (TIF) [file pone.0204241.s001.tif]

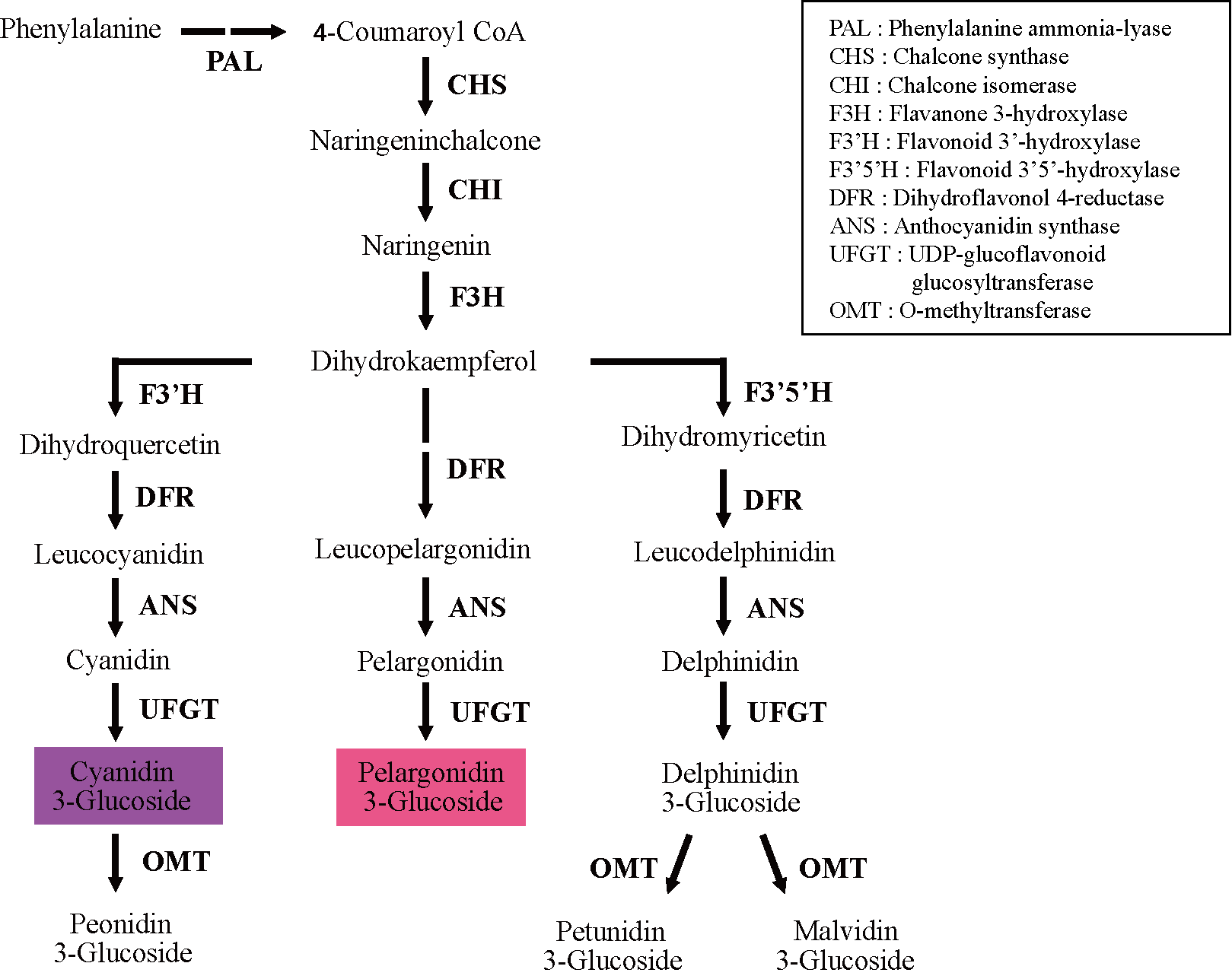

Supplement: S2 Fig — Enzymes for each step were abbreviated as indicated in the box. Two major anthocyanins pelargonidin 3-glucoside and cyanidin 3-glucoside present in radish roots are presented in colored boxes. (TIF) [file pone.0204241.s002.tif]

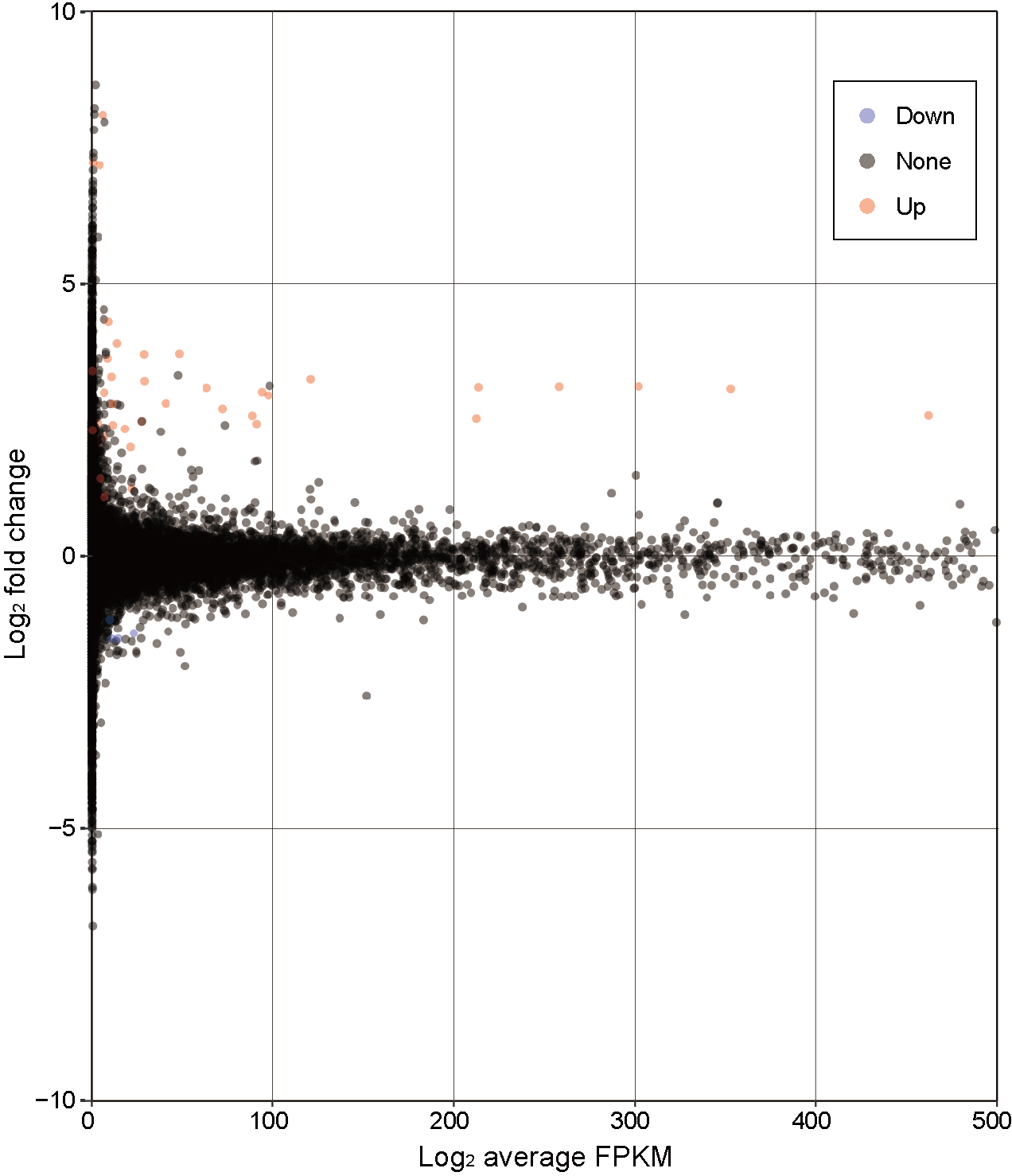

Supplement: S3 Fig — The FPKM and fold change averages were plotted. Significantly up- and downregulated genes were selected by the cutoff determined by Welch’s t-test and FDR < 0.05, and colored as indicated. (TIF) [file pone.0204241.s003.tif]

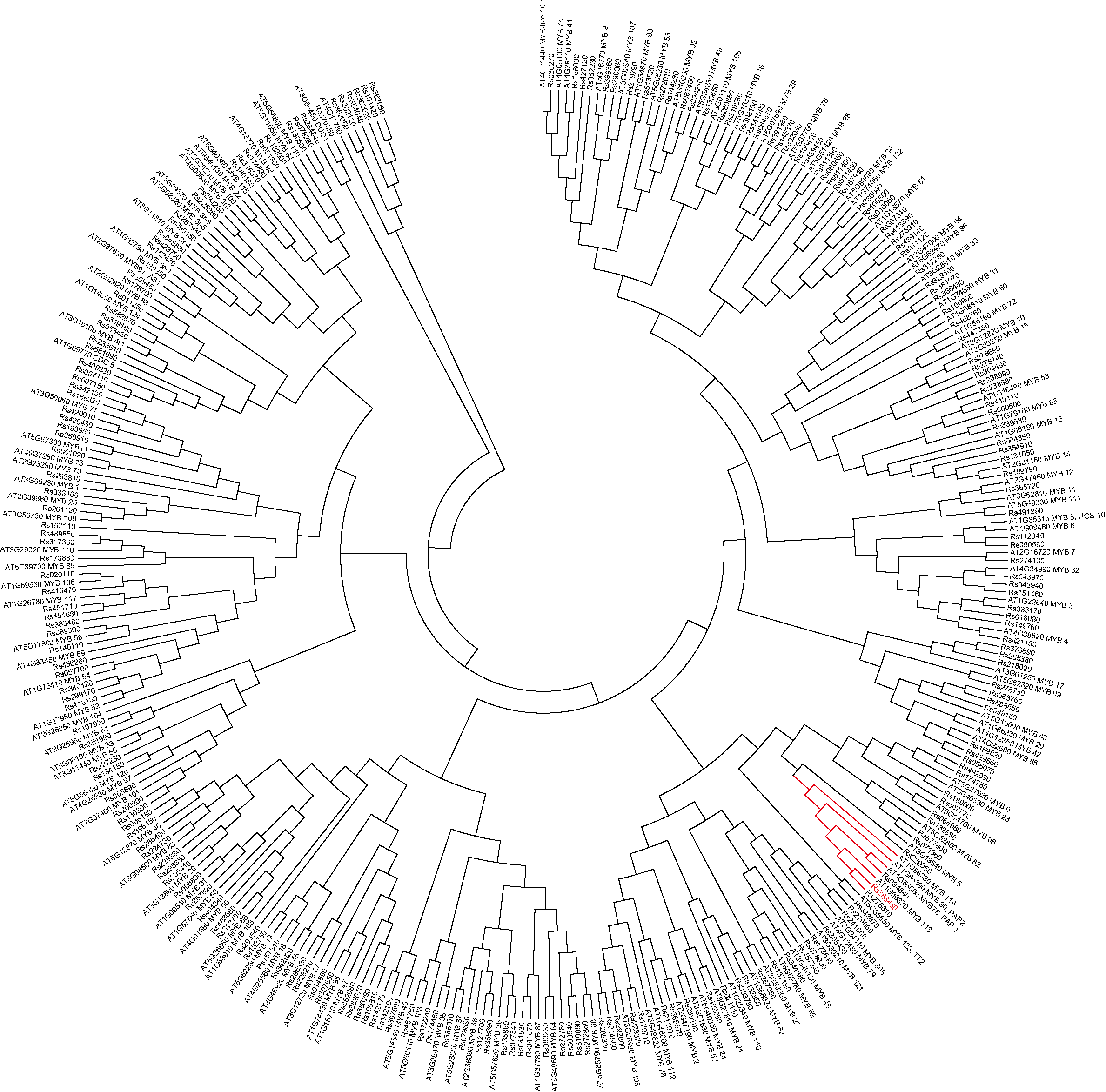

Supplement: S4 Fig — Red lines indicate the branch for anthocyanin accumulation regulators. RsMYB1 is denoted in red. HOS10, high response to osmotic stress 10; PAP1, 2, production of anthocyanin pigment 1, 2; TT2, transparent testa 2; AS1, asymmetric leaves 1; CDC5, cell division cycle 5; DUO1, duo pollen 1; AT4G17780, F-box and associated interaction domains-containing protein. (TIF) [file pone.0204241.s004.tif]

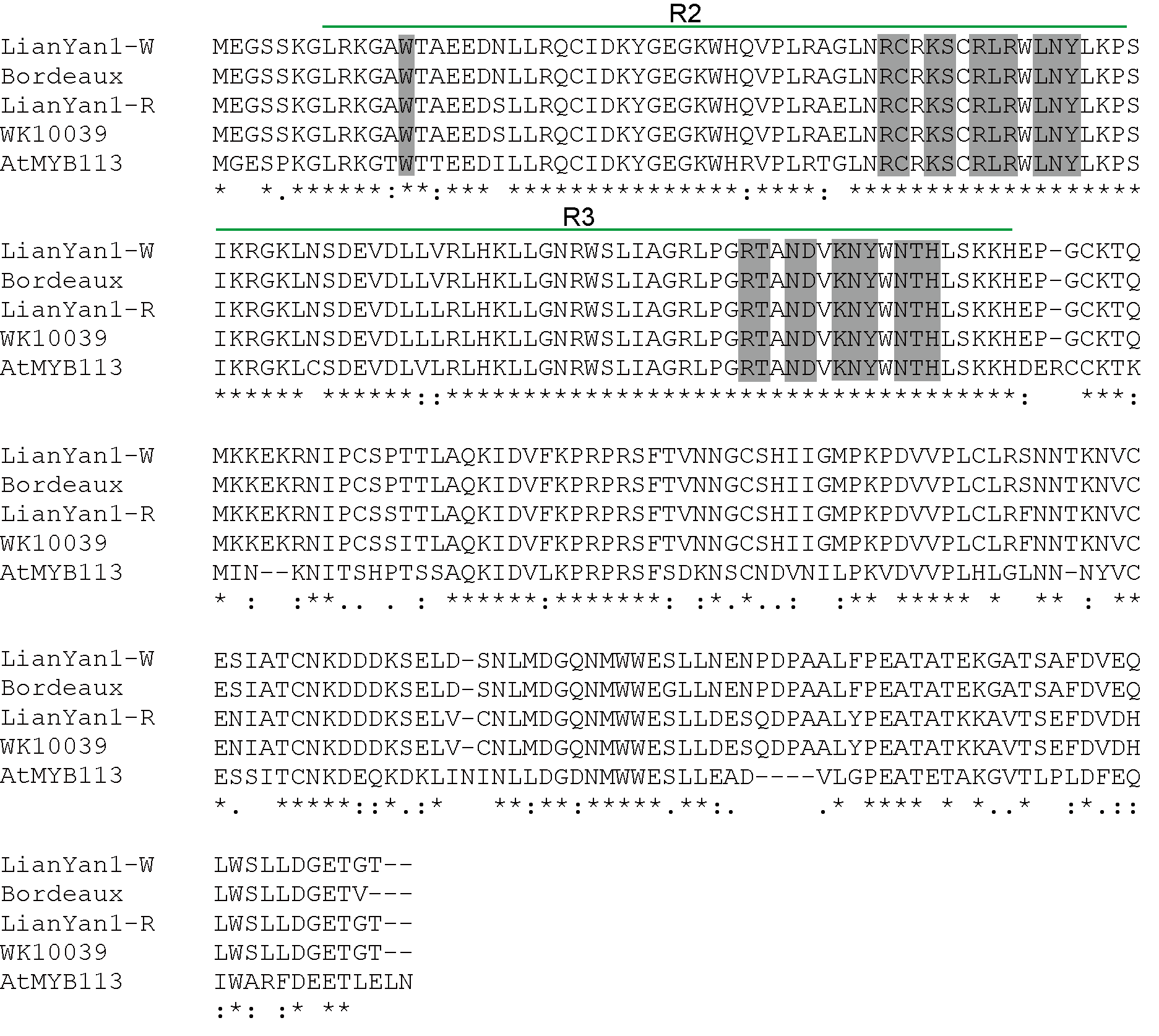

Supplement: S5 Fig — Translated RsMYB1 amino acid sequences were aligned to RsMYB1 from Bordeaux (AKM95888), WK10039 (Rs388430) and AtMYB113 (AT1G66370) by CLUSTAL-OMEGA. Green lines indicate the R2R3 domains of MYB DNA-binding proteins. Shaded boxes indicate putative DNA binding sites [27]. (TIF) [file pone.0204241.s005.tif]

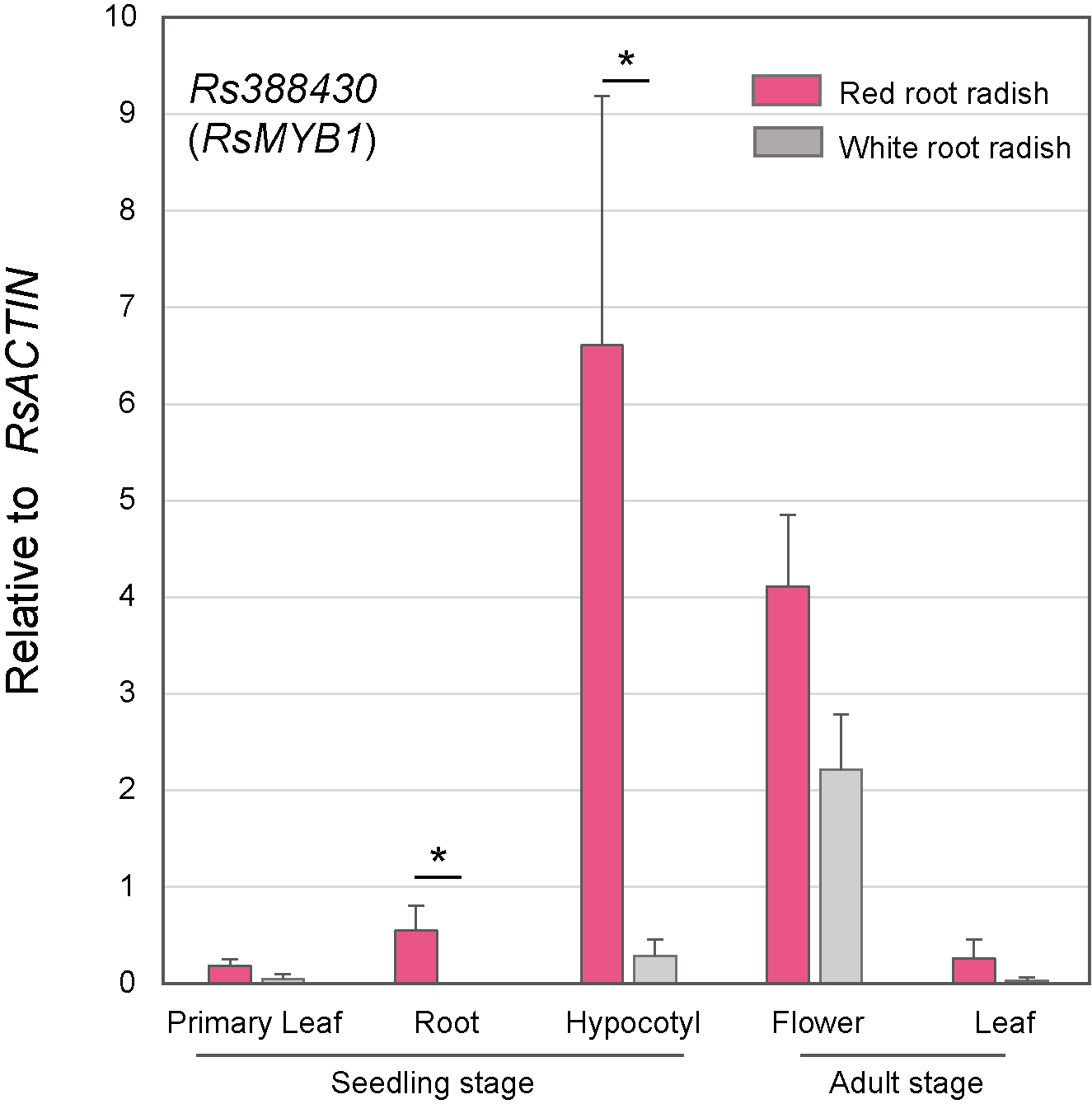

Supplement: S6 Fig — qRT-PCR was performed with three biological replicates for red and white root skin radishes, respectively. Data are means ± SD. * p < 0.05. (TIF) [file pone.0204241.s006.tif]

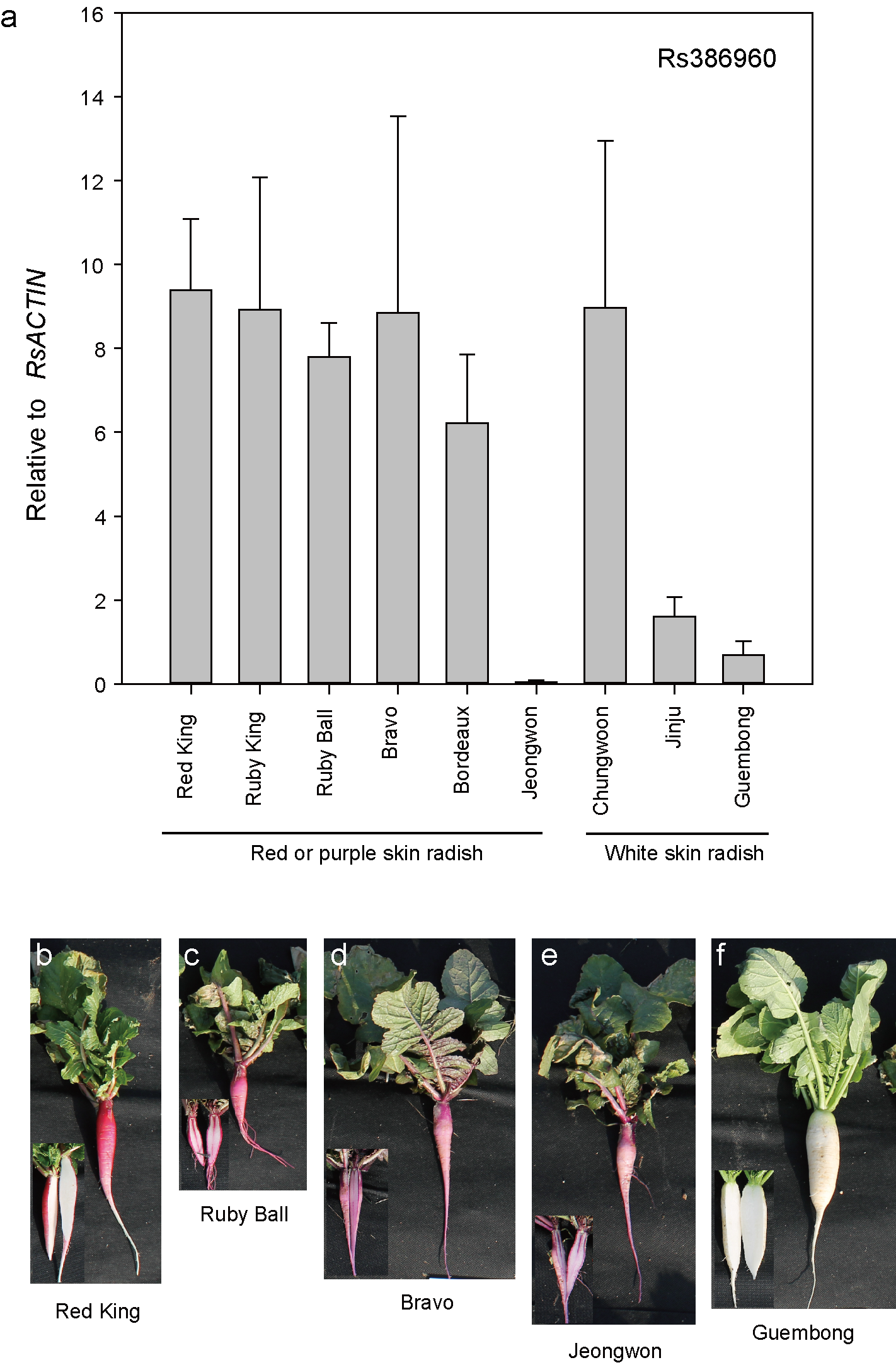

Supplement: S7 Fig — (a) qRT-PCR analysis of Rs386960. Error bars indicate the standard deviation of the mean from three biological replicates. (b-f) Four-week-old roots of some radish cultivars used for qRT-PCR in Fig 4b and (a). (TIF) [file pone.0204241.s007.tif]
